# Battery Electrode Engineering Enables Fast Charging LMFP Cells Without Carbon‐Coated Foil

**DOI:** 10.1002/advs.78510

**Published:** 2026-10-26

**Authors:** Rebecca Sellers, Daniela Proprentner, Amir Dahari, Steve Kench, Galo Paez‐Fajardo, Louis Piper, Gerard Bree

**Affiliations:** ^1^ WMG University of Warwick Coventry UK; ^2^ The Faraday Institution Didcot UK; ^3^ Polaron London UK; ^4^ Dyson School of Design Engineering Imperial College London London UK

**Keywords:** cathode, current collector, electrochemistry, electrode, lithium‐ion battery, materials science, microscopy

## Abstract

Lithium manganese iron phosphate (LiMn_x_Fe_1‐x_PO_4_, LMFP) has emerged as a promising Li‐ion battery cathode material due to its high voltage, thermal stability, and cycle life, yet its intrinsically low electronic and ionic conductivity limits high‐rate performance and thus usefulness in fast‐charge applications such as electric vehicles. Here, we evaluate key electrode design aspects, investigating the interplay between calendering density and current collector priming (carbon‐coating) on the fast cycling performance of LMFP cathodes. For electrodes on pristine aluminium, calendering to high density induced particle embedment into the foil (quantified via segmentation of cross‐sectional microscope images), reducing interfacial resistance while enhancing both rate capability and long‐term cycling stability. In contrast, performance of electrodes on C‐coated aluminium exhibited minimal sensitivity to densification. Surprisingly, at sufficiently high calendering pressure (achieving density ≥ 2.5 g cm^−3^), the carbon coating provided no advantage to high‐rate performance, in stark contrast with conventional understanding, and cells manufactured with pristine Al achieved fast charging from 10% to 80% SOC in just over 15 min, a key milestone for this chemistry. These findings establish a mechanistic understanding of how density and interfacial properties govern transport and electrochemical behavior in LMFP cells, providing practical guidance for optimized electrode structures.

## Graphical abstract

Slow charging inhibits the widespread adoption of electric vehicles, particularly for LMFP, a new low‐cost, high‐energy battery material. In this work, the authors report on electrode engineering approaches (optimized press density and current collector foil priming) to increase energy density and reduce LMFP charge time to just 16 min (10%–80% SOC).

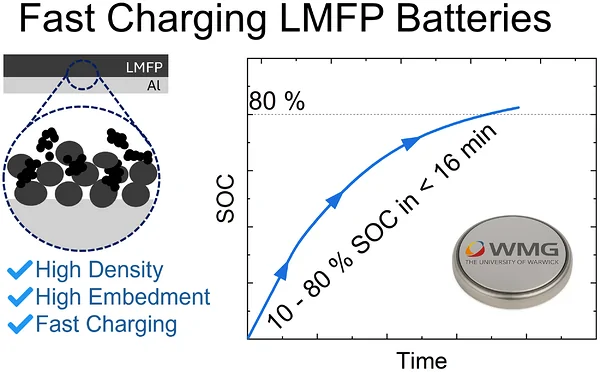

## 1 Introduction

LMFP has emerged as a promising cathode material candidate to address outstanding Li‐ion battery challenges such as improving energy density/lifetime, reducing production costs, and enhancing life‐cycle sustainability [1, 2, 3]. This is achieved through a combination of the high voltage of the Mn^2+^/Mn^3+^ redox couple (∼4.1 V) with the stability and cycle life of LiFePO_4_ (LFP), while avoiding costly raw materials (Ni and Co). The partial substitution of Fe with Mn (moving from LFP to LMFP) enhances energy density while mitigating the deleterious effects of heavy Jahn‐Teller distortion typical of LiMnPO_4_ (LMP) [4, 5]. The particular level of substitution chosen will heavily influence the electrochemical characteristics of the material, with high Mn content (≥ 80%) providing increased energy density but degrading kinetics and stability, while low Mn content (< 50%) providing an insufficient boost over LFP. Consequently, many academic studies adopt a medium Mn content of 50%–70%, achieving a balance of these factors [1, 2, 6, 7]. Nevertheless, LMFP inherently suffers from low electronic and ionic conductivity, limiting high‐power performance and requiring careful design of optimum particle and electrode microstructures to achieve sufficiently rapid Li^+^ and e^−^ transport [8, 9, 10]. At the particle level, LMFP is typically nanosized to minimize Li^+^ diffusion distances, and coated in conductive carbon at the synthesis stage to enhance electronic conductivity. At the electrode level, effective particle dispersion along with careful consideration of microstructure and formulation (conductive additive and binder content) is required to enable effective transport properties.

The electrode calendering process, a pressing/densification step typically carried out after drying, is designed to reduce electrode thickness and volume via compaction, thereby enabling greater energy storage capacity within a given volume [11, 12, 13]. To achieve this, the electrode is passed between two chrome rollers, experiencing compression and thus densifying the layer. The final, post‐calendering thickness/density is controlled via modulation of the calendaring “gap,” i.e., the distance between the two rollers (determining the pressure experienced by the electrode), along with line speed and temperature [14, 15]. Calendering can additionally improve electrode adhesion, cell lifetime, and kinetics via enhanced interparticle and interfacial contact [15, 16]. Given the poor conductivity of LMFP, microstructural and interfacial optimization is particularly critical, as the small particle size presents a challenge to a high level of compaction [17, 18]. Conversely, modern LFP particle design utilises advanced morphologies and size distributions to maximise packing efficiency [19, 20, 21]. As such, studies of LMFP typically utilise densities in the range 1.8–2.2 g cm^−3^ [1, 2], far lower than that of state‐of‐the‐art LFP (up to 2.7 g cm^−3^ industrially and > 3.0 in a recent academic report) [20, 21]. This remains a barrier to LMFP competitiveness, and bridging this gap requires the design of LMFP particles suitable for high compaction, along with optimization of the calendaring process itself.

Priming Al foils with a thin (typically ∼1 µm) carbon‐ based coating (C‐coating) has been shown to offer significant benefits to battery performance compared with pristine Al foils [22, 23, 24]. Various forms of this coating have been proposed, in which the film takes the form of carbon black [25], graphene sheets [26], or nanotubes [27]. These coatings reduce interfacial resistance between active and current‐collecting layers, providing a high surface area and highly conductive network, ensuring efficient electron transport. They can also provide benefits to long‐term performance associated with improved active layer adhesion and high‐voltage stabilization [28], and may open opportunities for aqueous processing of cathodes, acting as a barrier preventing corrosion of the Al foil by the alkaline slurry during coating [29]. C‐coated foils have demonstrated compatibility with both layered oxide [29] and phosphate cathodes [23, 24, 25]; however, few studies have focused on interactions with, and optimization of, LMFP cathodes [30]. While some advantage to both high C‐rate capacity and cell lifetime was observed, the effect was modest and dependent on carbon morphology (favoring graphene over nanotube), while the effect of electrode density was not investigated [24]. In contrast to these trends, there is ongoing demand for ever‐thinner, lighter‐weight current collectors. Indeed, the thicknesses of Al and Cu foils in typical commercial lithium‐ion batteries fell by 44% and 70%, respectively, in the period 1999–2021 [31]. Furthermore, the added processing step (deposition of the carbon layer) would likely add significant cost; indeed, current online prices reveal an approximately six‐fold increase in cost for carbon‐coated foils compared with pristine foils (Table S1, albeit at medium lab scale).

Thus, while the addition of a C‐coating necessarily adds dead mass and volume to a cell, it is often viewed as a necessary evil, particularly if fast cycling is an application requirement, e.g., fast charging of EVs, and academic/industrial interest in this area is growing [22]. Yet, the underlying mechanisms governing interfacial contact resistance and how it evolves with active material properties/morphology and, particularly, the electrode densification process, remain insufficiently understood. Furthermore, reports on analysis of the behavior of LMFP with C‐coated foils are very limited given the relatively recent and rapid adoption of this advantageous cathode.

In this study, we systematically investigate the simultaneous influence of electrode density (varied via modulation of calendaring gap) and current collector priming on the electrochemical performance of LMFP cathodes. In the case of LMFP on pristine aluminium, performance was heavily dependent on density, while on C‐coated aluminium, electrode densification had little effect, highlighting the influence of the current collector coating in mitigating interfacial limitations. This work establishes a mechanistic understanding of how electrode densification and interfacial properties collectively govern the transport and interfacial behavior of LMFP cathodes and clarifies the role of C‐coated foils with newer cathode chemistries. The findings provide practical guidance for optimising electrode design to enhance rate capability and long‐term cycling stability in next‐generation phosphate‐based lithium‐ion batteries.

## 2 Experimental

### 2.1 Slurry and Electrode Manufacture

The LMFP material utilized in this study (LiMn_0.6_Fe_0.4_PO_4_, LMFP60, Gelon LIB) was characterized in our previous study, including powder scanning electron microscopy (SEM) and X‐ray fluorescence analysis [1]. The slurry mixing and electrode manufacturing processes were described in a previous publication [2]. Briefly, LMFP, PVDF (5130, Solef Solvay), and carbon black (C65, Imerys) were dried at 60°C for 12 h under vacuum and mixed in a mass ratio of 93:3.5:3.5. Mixing was carried out using an Eirich EL01 mixer with N‐methyl‐2‐pyrrolidone (NMP, Sigma–Aldrich) solvent to achieve a solid content of 46.3 wt.%. The slurry was cast (ERICHSEN COATMASTER 510) onto pristine Al foil (15 µm, Avocet) and C‐coated Al foil (13 µm, Cambridge Energy Solutions) to a coat weight of 135 gsm (13.5 mg cm^−2^), before drying overnight under vacuum at 120°C. The electrodes were calendered at 80°C to a desired thickness/density via modulation of the roller gap, then punched into 14.8 mm discs and dried further at 120°C for 12 h prior to transfer into an argon glovebox.

Anodes were fabricated using WMG's Battery Scale‐Up Line (University of Warwick). The formulation was 95.25:1:1.5:2.25 (Graphite:CB:CMC:SBR), mixed using an Eirich EL1. The slurry was coated onto copper foil (8 µm, Avocet) using a Megtec reverse comma bar roll‐to‐roll (R2R) coater, dried, and calendered to a density of 1.4 g cm^−3^. The electrodes were punched into 15 mm discs and dried at 50°C for 12 h, achieving an N:P ratio of 1.05–1.10.

### 2.2 Electrode Characterization

Resistance probe measurements of calendered electrodes were carried out using a Hioki RM2610 46‐point system. A finite volume model (FVM) is used to determine both the volume resistivity of the coating and the interface resistance between the coating and the foil. For scanning electron microscope (SEM) analysis, a Hitachi IM4000 Broad Beam Ion Miller was used to prepare cross sections of the cathodes. Broad beam conditions were set to 6 kV acceleration voltage, 1.5 kV discharge voltage, and a 40° milling angle while milling for 80 mins. A Thermo Fisher Scios DualBeam was used for simultaneous imaging of backscattered, secondary electron, and in‐lens signals at 2 kV acceleration voltage and 0.2 nA beam current. The Thermo Fisher Maps software was used to collect up to 200 individual images per detector at a pixel resolution of 26 nm, which were then stitched together to approximately 700 µm‐wide maps of the cross sections covering the electrode height. Electrode heights were measured based on these images at ten locations of the imaged cross section. High‐resolution images were collected at a pixel resolution of 6.5 nm on a Thermo Fisher Scientific Helios 5 Hydra plasma focused ion beam (PFIB) at 2 kV acceleration voltage and 0.2 nA beam current. The electrode was segmented using the software Dragonfly.

X‐ray diffraction (XRD) measurements on the electrodes were performed in the Bragg‐Brentano mode using a Malvern Panalytical diffractometer (Cu Ka radiation). The data were collected between 5° and 80° (2θ) using a step size of 0.02°, and a dwell time of 200 s. To reduce fluorescence from Mn and Fe, the lower PHD settings of the detector were increased appropriately.

Hard X‐ray photoelectron spectroscopy (HAXPES) measurements of core regions were performed at the beamline I09 at Diamond Light Source using a photon energy of 2.2 keV with a pass energy of 200 eV, corresponding to a 250 meV energy resolution. The binding energies of HAXPES regions were referenced to the so‐called adventitious carbon (C 1s) peak at 284.5 eV.

Soft X‐ray absorption spectroscopy in the total electron yield (TEY) mode on the Fe L‐edge also took place at the Diamond beamline I09. To account for beam irregularities, the absorption signal was divided by the signal from a reference Cu‐coated mesh (I0) in the incident photon beam. The Fe L‐edge scans were background‐normalized. The XAS were energy‐calibrated via the valence band of Au foil using accordingly a photon energy excitation corresponding to the main peaks in the Fe L3‐edge.

### 2.3 Cell Assembly and Electrochemical Measurements

2032‐type full coin cells were assembled in an Ar‐filled glovebox (O_2_ and H_2_O levels < 0.1 ppm) using the as‐prepared LMFP electrodes as cathode, and graphite as anode, a polyolefin separator (Celgard H2325), and 120 µL of electrolyte consisting of 1 M LiPF_6_ in ethylene carbonate and ethyl methyl carbonate (EC:EMC = 3:7 by weight) with 2 wt.% vinylene carbonate (Solvionic). The cells were crimped using an MTI digital coin cell crimper. Three‐electrode “PAT” cells (EL‐cell), with 18 mm diameter electrodes and a Li reference ring, were assembled using 200 µL electrolyte.

Galvanostatic charge and discharge tests were carried out in a temperature‐controlled chamber at 25°C using Biologic BCS (coin cells) and VMP3 (PAT cells) systems, in conjunction with EC‐lab software. The cells underwent an initial 10 h wetting stage before 2 formation cycles at C/20 using a constant‐current protocol, cycled between 2.5 and 4.3 V. After formation, the cells underwent symmetrical rate testing at C/3, C/2, 1C, 2C, 3C, and 5C, then returned to C/3 (whereby 1C was defined as 160 mAh g^−1^ practical capacity). A constant‐current–constant‐voltage (CC–CV) charging protocol was employed, with the CV step terminating when the current decreased to < 10% of the CC value. Subsequent cycling was performed for 300 cycles at C/3 within a voltage window of 3.0–4.3 V under CC–CV charging, with diagnostic cycles at C/20 conducted every 100 cycles. Cell performance is expressed as specific gravimetric capacity (mAh g^−1^, based on cathode active mass) or specific volumetric capacity (mAh cm^−3^, based on cathode active layer volume including active material, carbon, binder, and pore). Electrochemical impedance spectroscopy (EIS) was carried out after cell formation, following C‐rate testing, and every 100 cycles thereafter. Measurements were performed at a fixed potential of 2.5 V with a 10 mV perturbation amplitude over a frequency range spanning 1 MHz to 10 mHz.

### 2.4 Statistical Analysis

Battery cell test data (voltage, current, time) were processed into capacity data utilising Biologic BT‐lab software inherent functionality. For coin cell testing, 3 cells were produced in each condition, with both the best‐performing (e.g. Figure 1) and the mean plus standard deviation (e.g. Figure S5) shown in this work. P‐values were utilized to indicate statistical significance using Origin 2026 software.

**FIGURE 1:**
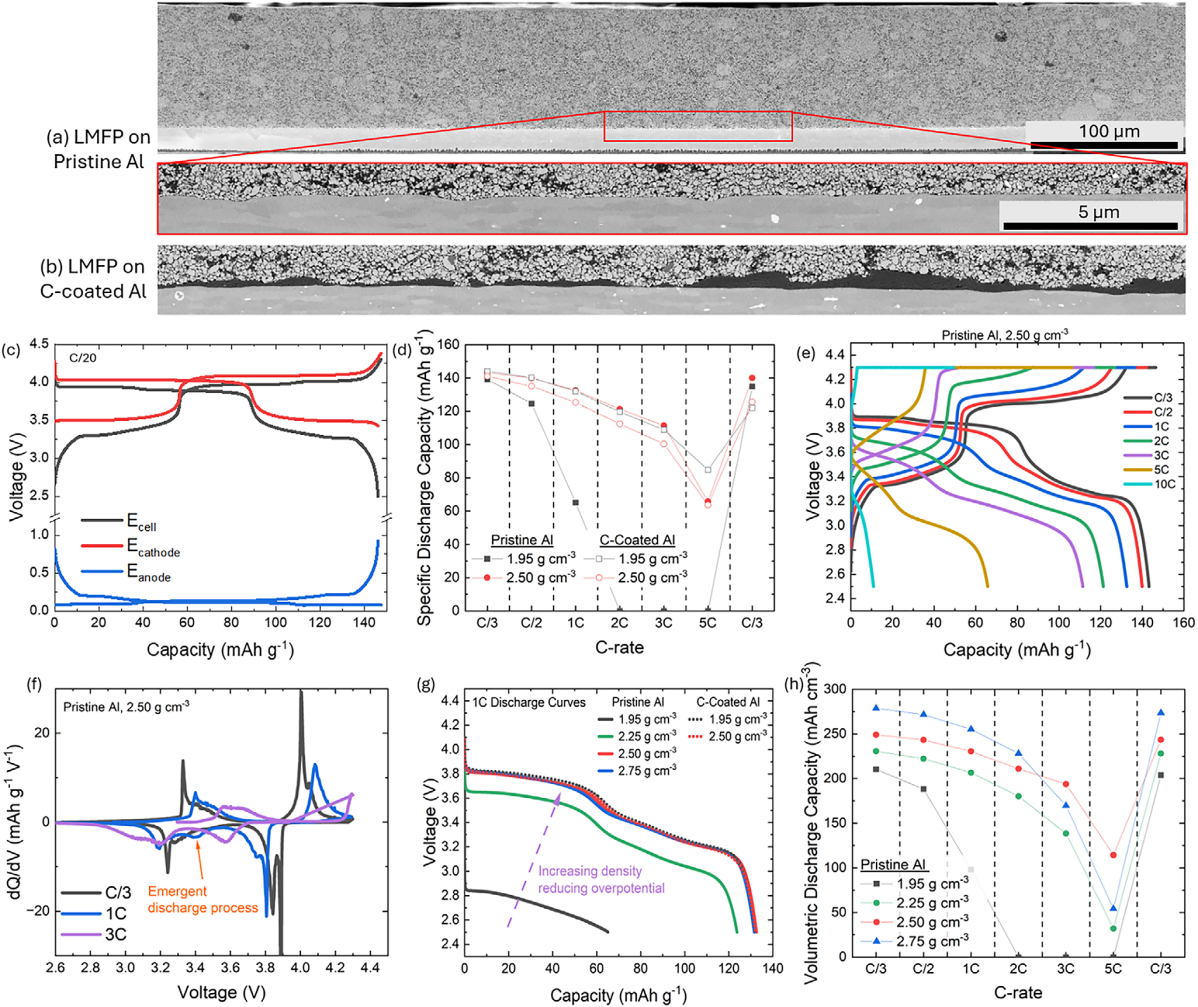
Backscattered electron images of an LMFP electrode on (a) pristine aluminium and (b) C‐coated aluminium, highlighting the interface between active and current‐collecting layers; (c) Voltage profile of a 3E LMFP//Gr cell cycled at C/20, tracking cell, cathode, and anode potentials. Rate capability testing results of LMFP∖∖Gr cells calendered to a variety of densities, comparing pristine and carbon‐coated aluminium current collectors. (d) Specific discharge capacity: 5 cycles were performed at each cycling rate; the capacity from the first cycle only is shown here for clarity. Capacity from all cycles is shown in Figure S4. (e) voltage profiles and (f) dQ/dV plots for pristine Al, 2.50 g cm^−3^. (g) discharge voltage profiles at 1C, comparing varying density and foil. (h) rate capability test expressed as volumetric discharge capacity. Data shown here are from representative cells; the average of all cells is shown in Figure S5.

## 3 Results & Discussion

The LMFP electrodes utilized in this study were manufactured to high active loading (93%), thickness (80 µm), and coat weight (135 gsm) to ensure commercial relevance. Figure 1a,b show cross‐sectional SEM images of electrodes on pristine and C‐coated Al foil, respectively. The interface of the active layer with the current collector is shown in higher magnification Figure 1a,b, for pristine and C‐coated foils, respectively. The C‐coating consisted of a layer of carbon black of thickness up to a maximum of approx. 1.4 µm, located between the Al foil and active layer, designed to provide enhanced electronic contact. Initially, the LMFP electrode was assembled into a 3E LMFP∖∖Gr cell to validate performance and ensure appropriate electrode balancing. Figure 1c shows the capacity–voltage profile of this cell during cycling at C/20, highlighting the individual contributions of the anode, cathode, and full cell. Here the advantage over more traditional LFP is clear, whereby the LMFP cathode exhibits two distinct redox plateaus at approximately 3.4 and 4.1 V, corresponding to the Fe^2^^+^/Fe^3^^+^ and Mn^2^^+^/Mn^3^^+^ redox couples, respectively [16]. A high capacity of > 147 mAh g^−1^ confirms the quality of the active materials and the produced electrodes, while the LMFP exhibits expected behavior (TM oxidation & minimal surface degradation) during cycling, as confirmed via X‐ray analysis of electrodes in the pristine and charged states (Figure S1). Material quality and resultant cell performance were further demonstrated via long‐term testing of a pouch cell made with these electrodes (Figure S2).

To evaluate the impact of the C‐coating and its interaction with electrode density, LMFP∖∖Gr full coin cells were assembled using LMFP electrodes on pristine and C‐coated foils, and calendered to a variety of densities. Density is a critical electrode design parameter, impacting Li^+^ transport within both the active phase itself and the pores of the electrodes, significant factors in determining the cell response to high current loads. The range studied here, from 1.95 g cm^−3^ (light densification, 42% porosity) to 2.75 g cm^−3^ (heavy densification, 18% porosity), covers values associated with state‐of‐the‐art and next‐generation LFP (∼2.3–2.7 g cm^−3^) [21, 32] along with reported values for LMFP in literature (which are typically lower), ∼ 2.0–2.2 g cm^−3^ [1, 2, 3]. The method of calculation of porosities and densities is shown in the SI. To achieve sufficiently high pressure/line load to produce higher density electrodes (≥ 2.5 g cm^−3^), it was necessary to calender only a small electrode width (< 2 cm). Even under such extremes, the densification process itself is not expected to induce any compositional changes in the electrode, verified here via XRD analysis before and after calendering (Figure S3).

A rate capability test was performed on these cells, with results shown in Figure 1d. At the low C‐rate of C/3, capacity was not affected by either electrode density or current collector, with all electrodes exhibiting a discharge capacity of approx. 140 mAh g^−1^. However, at higher C‐rates, performance diverged significantly. The electrode coated on pristine Al at a density of 1.95 g cm^−3^ (minimal densification) exhibited a pronounced decrease in discharge capacity, ultimately showing almost no measurable capacity at 2C. In contrast, the equivalent cell utilising C‐coated foil exhibited good performance at even very fast rates (85 mAh g^−1^ at 5C). This enhanced high‐rate capacity is consistent with the traditional understanding of the role of a C‐coated foil, and points to the importance of this interface in determining performance characteristics [2, 33]. More surprising, however, is how this enhancement evolves with changing calendaring conditions/densification. At a higher density of 2.50 g cm^−3^, both the pristine and C‐coated cells performed comparably regardless of cycling rate, e.g., 66 and 64 mAh g^−1^ at 5C, respectively. In fact, the pristine Al exhibited a small performance advantage in the medium cycling range (C/2–3C); however, analysis of cell‐to‐cell variance (Figure S5) indicated that this difference was not statistically significant. In summary, the high‐rate performance on pristine Al was heavily dependent on the level of densification, while that on C‐coated Al was largely independent of density. When densification was sufficiently strong (2.50 g cm^−3^), the benefit to the C‐coated was eliminated or even reversed, in stark contrast with traditional understanding of these foils.

The trend in capacity with C‐rate can be better understood via investigation of voltage profiles and selected dQdV plots (shown in Figure 1e,f, respectively, and Figure S6). Here, an increase in polarization/overpotential at high C‐rates was observed, elevating charge and depressing discharge plateaus, leading to premature reaching of 2.5 V and thus limiting discharge capacity. Two further phenomena are apparent from the dQdV. First, in addition to the polarization‐induced peak voltage shift, the peaks also broaden, indicating increased levels of electrode heterogeneity at higher rates. Second, a further peak emerges in the discharge process, most clearly visible in the 1C curve at approx. 3.4 V. This has been observed for LMFP previously, although a clear mechanism has yet to be conclusively identified [1, 34, 35]. These factors act to reduce cell performance at faster rates; however, this is alleviated at higher electrode density. Figure 1g shows the discharge curves for cells of varying density, comparing pristine and C‐coated foil. The curves overlap almost exactly when C‐coated foil was employed, maintaining good capacity and high discharge voltage. With pristine Al, however, low density resulted in a greatly depressed discharge voltage. When density was increased to 2.50 g cm^−3^, the curve overlaps with those of the C‐coated foil, demonstrating that C‐coating provided no advantage to either capacity or average voltage when densification was sufficient.

When considering variations in electrode density, it is more instructive to interrogate the volumetric capacity (as opposed to gravimetric) as this accounts for the space‐saving nature of higher densities and neatly demonstrates the trade‐off between electrode densification and transport kinetics. Figure 1h shows rate capability plotted in terms of volumetric discharge capacity in mAh cm^−3^. Further medium‐ and high‐density electrodes (2.25 and 2.75 g cm^−3^) on pristine Al were produced to better elucidate performance trends. At low C‐rate, capacity followed a clear trend with density, with 2.75 g cm^−3^ providing the highest performance due to efficient packing of active material. However, as the C‐rate increased, pore charge transport effects dominated (seen via a crossover at 3C), limiting ionic accessibility [36, 37], and the highest 5C performance was achieved at a lower density of 2.50 g cm^−3^. These findings highlight the importance of balancing mechanical densification with electrolyte transport properties to achieve optimal rate performance for a given application. Nevertheless, the higher‐density electrode provided a clear advantage at all but the fastest rates, and the non‐viability of low‐density electrodes is clearly shown. This finding therefore poses both material synthesis challenges (designing LMFP of high tap density) and equipment design challenges (achieving high calendaring pressure) to achieve high density over a large electrode roll width while avoiding symptoms of overcalendering (cracking/delamination) [38]. While utilising C‐coated foil did improve low‐density performance at fast rates (Figure S7), little benefit was observed under most reasonable processing conditions.

The notable correlation of cell performance with LMFP electrode densification warranted further investigation. To gauge how the electronic resistance of the electrode evolved, interface (between active and current‐collecting layers) resistance measurements were performed on electrodes of varying density on pristine and C‐coated Al (Figure 2a). A dramatic trend was observed in the case of pristine Al foil. From a mean value of 10.4 Ω cm^2^ at a density of 2.15 g cm^−3^, an exponential decay was exhibited, reaching a value of 0.21 Ω cm^2^ at 2.7 g cm^−3^. The effect is very different when C‐coated foil was utilized, whereby contact resistance was low (< 0.2 Ω cm^2^) across all densities, highlighting the beneficial role of the carbon coating when electrodes were only lightly calendered. Crucially, at densities ≥ 2.5 g cm^−3^, the pristine Al interface resistance approached a comparably low value, negating the advantage of the C‐coating.

**FIGURE 2:**
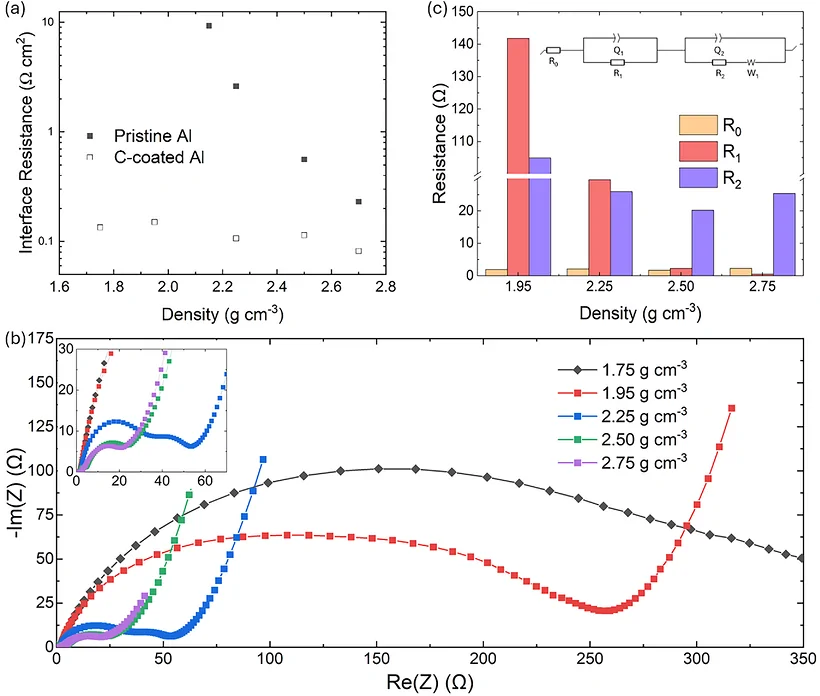
Characterization of electrodes and cells of varying density on pristine Al foil. (a) Active‐layer/Current collector interface resistance obtained via 46‐point resistance probe measurement on as‐manufactured LMFP electrodes on pristine and C‐coated Al (note: measurements of electrodes on pristine Al of density < 2.15 cm^−3^ were not possible due to extremely high resistance values). (b) EIS spectra of LMFP∖∖Gr coin cells recorded after formation, showing an inset zoomed plot of the high‐frequency region. (c) Extracted resistance values obtained using the inset equivalent circuit.

It is important to note that the resistance probe measurement considers only electronic transport, while battery operation of course relies on both electronic and ionic transport. To investigate sources of cell impedance in a practical electrochemical set‐up, EIS measurements were performed in LMFP//Gr full coin cells following formation (Figure 2b), with the corresponding fit shown in Figure 2c using the inset equivalent circuit model. The spectra consisted of two depressed semicircles, overlapping to varying degrees but most clearly resolved in the spectra for 2.25 g cm^−3^. The R_0_ component obtained from EIS reflects the ohmic resistance of the cell, encompassing contributions from the cell casing and connectors, although typically dominated by electrolyte in the bulk (i.e., excluding transport in the porous electrode) [39]. R_0_ exhibited a slight increase at higher electrode density, the source of which is unconfirmed but may reflect damage to the Al foil under high calendaring pressure.

The attribution of the first high‐frequency feature in EIS spectra (R_1_) has been the subject of considerable debate within academia. Several studies have proposed that this response originates from contact resistance effects at the electrode/current collector interface, where insufficient contact or surface oxide layers can dominate the high‐frequency impedance behavior [40, 41]. Conversely, other reports have assigned this feature to Li^+^ diffusion within the anode solid electrolyte interface (SEI), particularly in systems where SEI formation and evolution significantly influence the interfacial impedance [42, 43]. Distinguishing between these two contributions remains challenging, as both can exhibit similar characteristics at frequencies [43, 44]. The high R_1_ values observed at low densities were consistent with the resistance probe measurements, indicating that the light calendering conditions did not result in the formation of good interfacial contact. However, at higher densities, R_1_ decreased greatly, again mirroring resistance measurements. This clearly confirms that the dominant phenomenon responsible for this EIS feature is an electronic interfacial resistance, rather than SEI (which would not be expected to vary with cathode density). Clearly, achieving appropriate interfacial contact via sufficient densification is a key prerequisite for good cell performance.

Charge transfer resistance (R_2_) reflects electrochemical reactions at the active material/electrolyte interface, including contributions from both the cathode and anode side. R_2_ exhibited significant dependency on LMFP layer density, initially decreasing with density, reaching a minimum at 2.50 g cm^−3^, beyond which it again increased. The source of the initial decrease is unconfirmed but has been observed previously [16], and may reflect the very poor interparticle and interfacial contact at low density, affecting even the medium‐frequency impedance. The increase of R_2_ at higher density of 2.75 g cm^−3^ is consistent with the expectation that the available LMFP surface area is reduced.

The question remains as to how the interface morphology evolves with level of densification to give rise to the dramatic changes in electrode properties. The extremely small particle size of the LMFP material would be expected to dramatically influence interactions with the current collector, and thus the properties of the interface. Lessons learned from other materials of larger particle size and differing kinetics may not apply, and thus design rules require updating [37]. Here, the underlying mechanisms behind the source of active layer/foil interfacial contact resistance were investigated via cross‐sectional SEM of as‐manufactured electrodes. The thicknesses of the active layers decreased with increasing density, from 75 to 50 µm (full height SEM images are shown in Figure S8), consistent with in‐line metrology and indicating minimal “relaxation” of the layers after densification. Figure 3a shows SEM images of the interface region of LMFP electrodes of varying density. The low‐density electrode exhibits a visibly high degree of porosity and minimal physical contact between active layer particles and the foil, likely impeding electron transport and contributing to increased interfacial resistance. As the level of densification was increased (via reduction of the roller gap), reaching a maximum density of 2.75 g cm^−3^, the porosity was visibly reduced, and the SEM image indicated clear evidence of active material embedment/“biting” into the foil (Figure 3b). This effect has been established for other active materials previously, but notably was not observed for LMFP [30], which we attribute to a variance in particle morphology between this study (small single or slightly agglomerated crystals) and previous (larger porous secondary particles). The latter morphology can accommodate high calendaring pressure via the closing of pores within particles, while the former cannot, and thus embeds into the foil. To quantify these trends, the SEM images were segmented into LMFP, carbon‐binder domain/pore, and Al foil phases (Figure 3a). This enabled quantification of the fraction of the foil surface that contained embedded/contacting LMFP particles (Figure 3c). This “embedment ratio” varied from 28% at 1.95 g cm^−3^% to 83% at the highest achievable density of 2.75 g cm^−3^. The trend in interfacial contact resistance (and the concurrent dramatic trend in cell performance) can be ascribed to this simple mechanical feature of battery electrodes and would occur for other active materials under sufficient pressure; e.g., layered oxides and spinels exhibit higher hardness and Young's modulus. Given the small particle size, the embedment distance is quite low (< 100 nm) compared with total foil thickness (15 µm), and thus significant damage to the integrity or the conductivity of the foil is not expected, even when double‐sided electrodes are considered. While the specific values for interfacial resistance may be influenced by the particular characteristics of the powder evaluated here (particle morphology, Mn content), the mechanism would be expected to be general. Furthermore, commercially available LMFP materials have tended to adopt the approach of individual or slightly agglomerated single crystals highlighted here [1]. In summary, a sufficient level of LMFP electrode densification provides sufficient pressure to achieve good embedment and interfacial contact, thus eliminating the performance gap between pristine and C‐coated foils.

**FIGURE 3:**
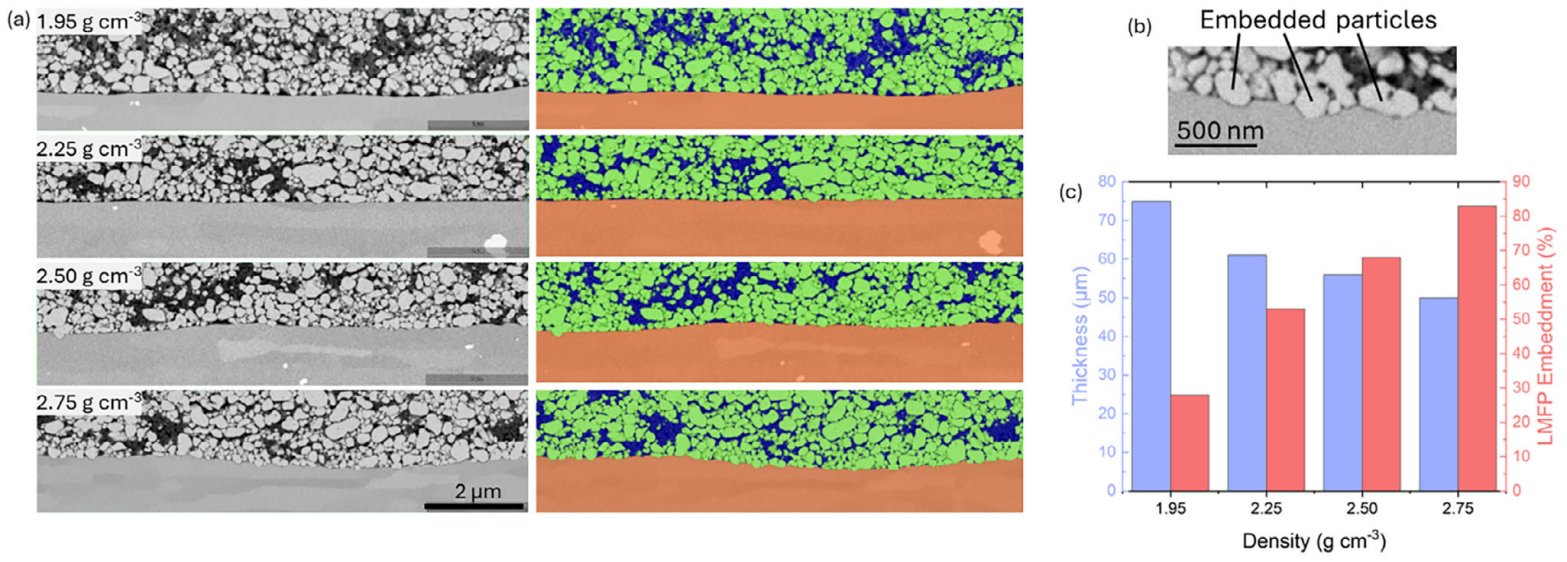
(a) Cross‐sectional backscattered electron images of LMFP electrodes of four densities in the range 1.95–2.75 g cm^−3^, along with the same images segmented into LMFP, CBD+pore, and Al foil phases. (b) High‐magnification image showing particle embedment into the Al foil in the electrode of density 2.75 g cm^−3^. (c) Electrode thickness and embedment % as a function of calendered density.

Alongside the effect on cell rate capability, the influence of LMFP density on long‐term stability was evaluated via repeated cycling of LMFP//Gr full coin cells (specific capacity shown in Figure 4a, and energy density in Figure S11) at C/3 for 300 cycles. The electrode of density 1.95 g cm^−3^ exhibited the lowest initial capacity and the most significant capacity loss after 300 cycles (61%). Both initial capacity and stability increased at higher density, up to 138 mAh g^−1^ and a loss of just 7% for the cell of density 2.75 g cm^−3^. Figure 4b,c show voltage profiles from selected points during the cycling ageing, for the lowest and highest density, respectively (profiles of the other densities shown in Figure S12). The 1.95 g cm^−3^ electrode exhibited poorly resolved features from the start, and a significant fraction of the overall charge capacity arises from the CV step, indicating sluggish kinetics. Such a situation would be expected to induce high electrode‐level heterogeneity, driving extreme localized parasitic reactions such as CEI‐forming electrolyte decomposition and compounding Mn dissolution [45, 46]. As such, the kinetics for the lowest density continue to worsen during cycling in a negative feedback loop, catastrophically impacting capacity. This kinetic‐dominated degradation mechanism of the low‐density electrode is further confirmed through regular (every 100 cycles) diagnostic cycling at C/20. The 1.95 g cm^−3^ electrode dramatically recovered to 135 mAh g^−1^ after 300 cycles (shown more clearly in Figure S13), confirming that Li inventory loss was not the major degradation mode. Conversely, the highest density electrode exhibited a low polarization, short CV step, and thereby maintained high capacity throughout cycling. This trend is in stark contrast with cells manufactured with a C‐coating and tested in a similar manner, whereby no effect of density on lifetime was observed (Figure 4d), even for uncalendered electrodes of just 1.75 g cm^−3^. Again, C‐coated foil provided an advantage only at low densities, which was eliminated via sufficient calendering of coatings on pristine Al foil.

**FIGURE 4:**
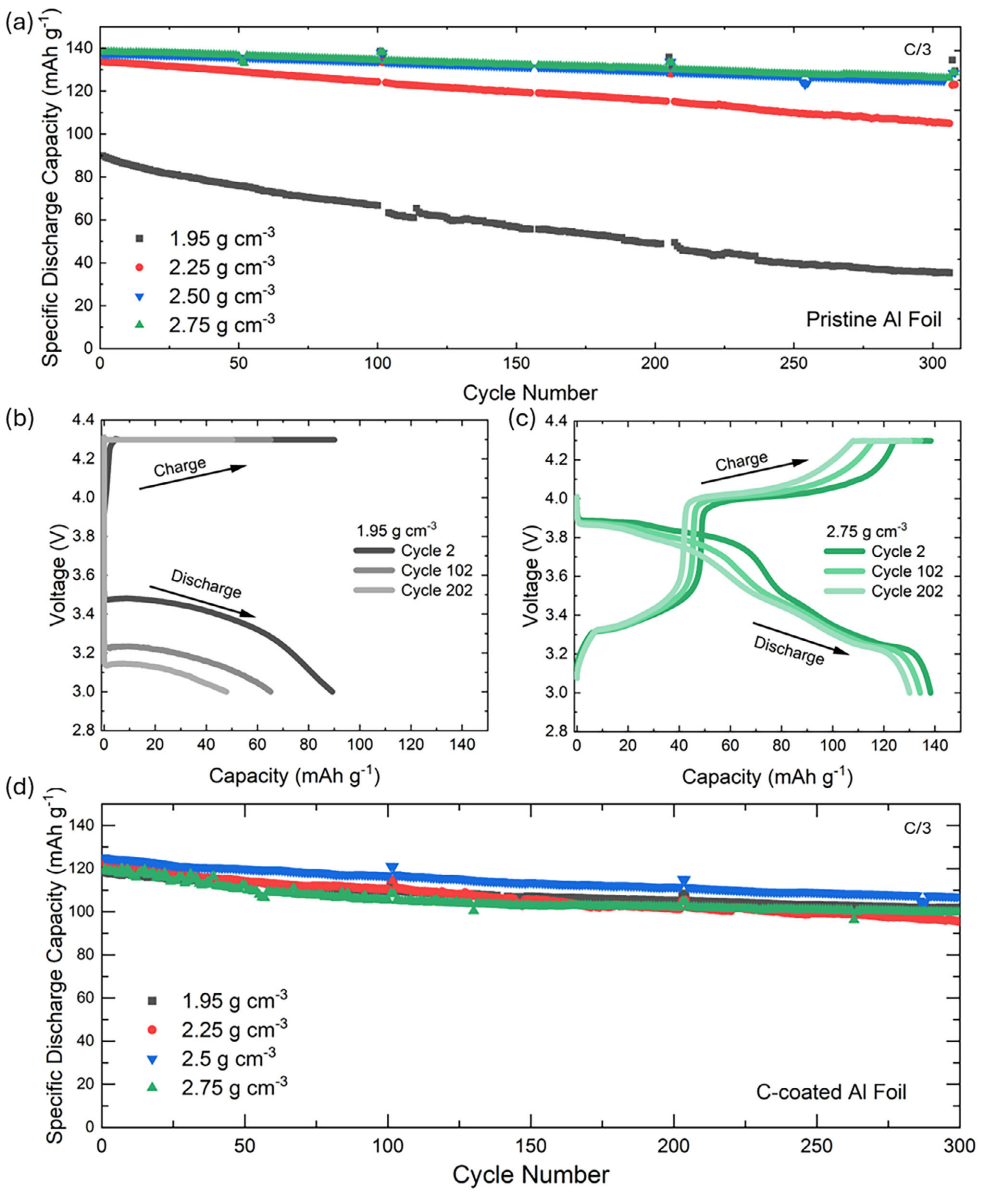
(a) Long‐term cycling of LMFP∖∖Gr full coin cells at C/3 (with diagnostic cycles at C/20 every 100 cycles), utilising LMFP electrodes of different densities coated on pristine Al foil. Voltage profiles over the course of cycling for (b) 1.95 g cm^−3^ and (c) 2.75 g cm^−3^. (d) Long‐term cycling of similar cells manufactured with C‐coated foil and cycled at C/3. For long‐term cycling tests, a voltage range of 3.0–4.3 V was chosen to better align with commercial cell operation, which limited depth of discharge in cases of high polarization (the effect of voltage range is shown in Figure S9). Coulombic efficiency data is shown in Figure S10.

The source(s) of the degradation in cell kinetics were evaluated via EIS. Figure 5a shows spectra obtained from the 2.25 g cm^−3^ cell (as an example density) at regular points during ageing. A gradual increase in total impedance, associated with growth in both semicircles, is visible. Figure 5b–d show the evolution of R_0_, R_1_, and R_2_ values for each density during ageing, extracted from fitting of EIS spectra. R_0_ contributed little to total impedance, with only minor changes during ageing. R_1_, ascribed largely to interface/contact resistance, increased for all cells, though far more dramatically for those of lower density. This may be attributed to a gradual reduction in embedment ratio as the cell was repeatedly charged and discharged (with associated LMFP expansion and contraction). Ex situ SEM analysis of an aged electrode (Figure S14) exhibited no obvious signs of degradation at the electrode level; however, analysis via image segmentation demonstrated an increase in the tortuosity of the active phase (Figure S15), implying disadvantageous morphological evolution. It should be noted that, while R_1_ was clearly dominated by contact resistance in the pristine cell, other high‐frequency elements such as SEI/CEI would be expected to grow with time and may come to play a larger role, although one would not expect these to be influenced by electrode density. R2 exhibited similar trends to R1, linked to degradation of transport and charge‐transfer properties, associated with defect formation, contact loss, and mechanical strain within the electrode [47]. The effect was most severe in low‐density cathodes, as their weaker particle connectivity and compromised structural integrity facilitate faster degradation under repeated cycling. Clearly, the use of appropriately densified LMFP electrodes was extremely effective at suppressing degradation mechanisms.

**FIGURE 5:**
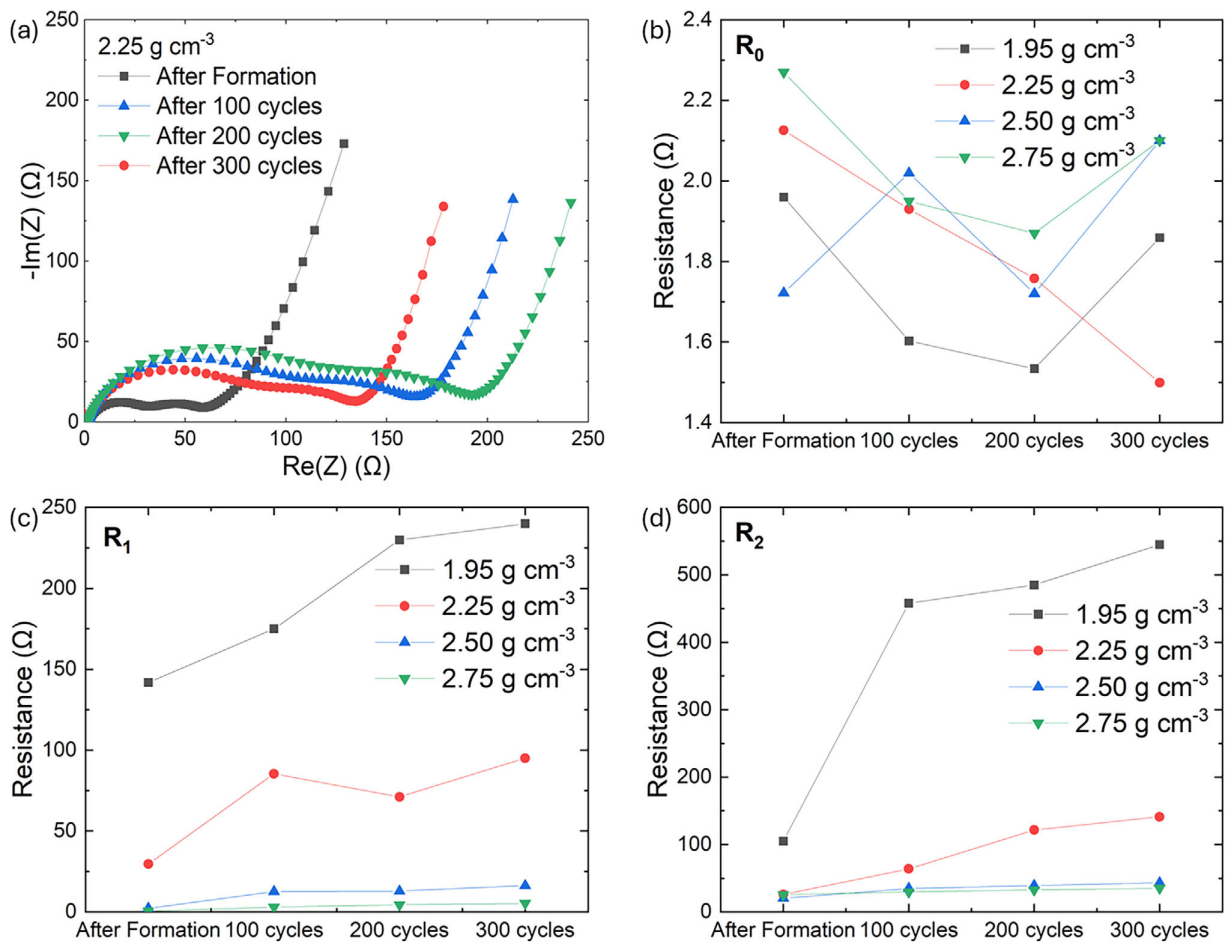
EIS spectra of LMFP∖∖Gr cell obtained at specified points during long‐term cycling. Evolution of resistances extracted from fitting of EIS curves with cycling at different densities: (b) R_0_, (c) R_1_ and (d) R_2_.

A key consideration with electrode density is the trade‐off between high volumetric density and fast charge capability. Highly porous electrodes are typically viewed as more favorable for fast charging due to rapid ion transport through the pores, highlighted by the rate capability test in Figure 1e [48, 49]. However, this test utilized a symmetric protocol, performing both fast charging and fast discharging, and thus capacity was limited by both phases of cycling, while for applications such as EVs fast charging is far more critical [2]. Therefore, a fast charge/slow discharge test (aligned with ISO 12906) was performed on LMFP∖∖Gr cells of selected densities, in which the charge rate was varied in the range C/3–10C, while the discharge rate was held constant at C/3 (Figure 6a). In general, capacities were higher than in the symmetric rate test, with 2.70 g cm^−3^ exhibiting a discharge capacity of 100 mAh g^−1^ after charging at 10C. While the worse performance of the 1.95 g cm^−3^ cell is not surprising, the observation of such excellent fast‐charge capability at high density perhaps is. Fundamentally, the critical value to the EV consumer is charge time, with charging from 10% to 80% SOC in < 15 min an industry‐wide goal [50, 51], with current models approaching that value (Table 1). Also shown in Figure 6a is the time taken to bridge this gap (secondary y‐axis). As charging current was increased, the charge time of course fell, reaching a plateau, reflecting the minimum charge time of which the cell was capable. A density of 2.70 g cm^−3^ exhibited the fastest charge times of the three densities, reaching 16 min under application of 5C. The favorable kinetics at high density enabled more capacity to be delivered during the constant‐current phase, instead of the subsequent, lower current, constant‐voltage phase (Figure 6b and Figure S16). Of course, this straightforward test provides only little information on any degradation induced during fast charging, and indeed the 2.70 g cm^−3^ cell did not recover its full capacity after returning to C/3, which can be attributed to Li plating during charging at 10C. Similarly, it does not at all consider system‐level effects such as maximum safe temperatures or infrastructural limitations. Nevertheless, such a short charge time for LMFP electrodes of high areal capacity and active loading, enabled via engineering of the active layer/current‐collector interface, represents a significant milestone in the development of this material.

**FIGURE 6:**
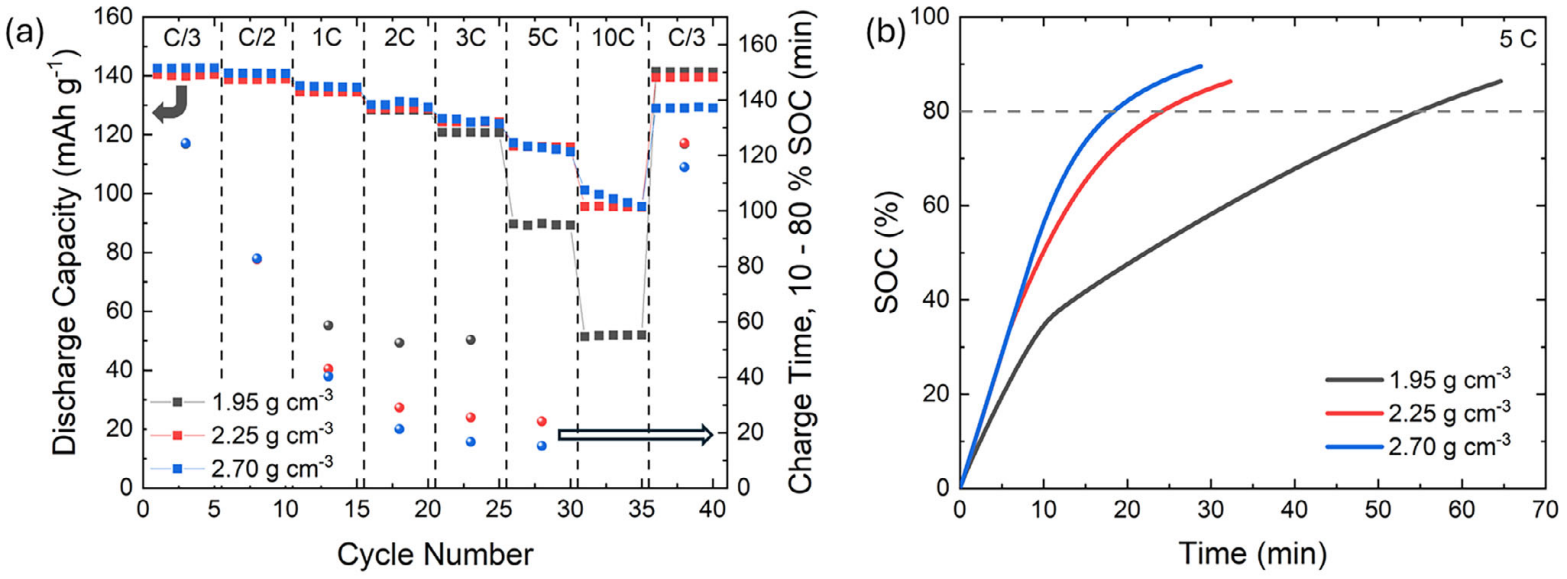
(a) Asymmetric rate capability test (CCCV charging, CV to 10% of initial current) of LMFP//Gr coin cell, maintaining a discharge current of C/3. The primary y‐axis shows capacity extracted during discharge, while the secondary y‐axis shows the time taken to charge from 10% to 80% SOC. SOC values are defined relative to a rated capacity of 140 mAh g^−1^. Omitted charge time data points indicate that the cell did not reach 80% SOC before the charge cutoff current was reached (e.g., 1.95 g cm^−3^ at 5C). (b) Cell SOC as a function of time during fast charging at 5C, comparing LMFP electrodes of three densities.

**TABLE 1:** Fast charge specifications of selected recent EVs. Data taken from Ref. [52].

Model	Cathode chemistry	Manufactured year	Fast charge C‐rate	Charge time, 10%–80% SOC (min)
Tesla model 3	LFP	2025	1.8C	24
Zeekr 7x long range RWD	NMC	2026	2.7C	16
Peugeot e‐2008	NMC	2024	1.4C	32
Audi e‐tron GT quattro	NMC	2025	2.8C	16
BYD SEALION 7 91.3 kWh AWD excellence	LFP	2024	1.8C	24

## 4 Conclusion

The development of optimized LMFP electrodes and cells represents a significant opportunity for improving the energy density, safety and lifetime of commercial Li‐ion batteries. This study reveals the impact of electrode densification level (modulated via control of roller gap and thus induced pressure) and current collector priming on the electrochemical performance of high coat weight and active loading LMFP cathodes. When utilising pristine Al foil, performance (high‐rate capacity and lifetime) was heavily dependent on the level of densification, while C‐coated Al foil provides good performance independent of this. Notably, calendering to a sufficiently high density on pristine Al removed the advantage of the C‐coating to both high‐rate cycling and lifetime testing. This thereby improved volumetric energy and cost via the higher density and removal of the inactive C‐coating. The active layer/current collector interface was a key factor in performance determination, and SEM and EIS analysis revealed that increasing electrode density promoted embedment into pristine Al, significantly reducing interfacial resistance, as confirmed by resistance probe measurements and EIS. High‐density (2.75 g cm^−3^) LMFP electrodes provided 93% capacity retention after 300 cycles and reduced battery charge time (10%–80% SOC) to < 16 min, a major marker for EV applications. While this sets a marker for what is possible for LMFP electrodes, it does not establish other potential limitations such as Li plating or brittleness caused by overcalendering (which may prevent electrode winding in cylindrical cell manufacture). These effects must be investigated and addressed to realise the true potential of fast‐charging LMFP cells.

The response of other active materials (NMC, LNMO) to high densification will depend on both hardness and morphology, whereby hard single crystals will embed effectively, while polycrystalline structures may be more likely to crack prior to embedment, degrading performance.

Looking ahead, high‐density engineered electrodes may yield even greater benefits to high‐Mn LMFP (given the lower intrinsic electronic conductivity) and thus be an enabler for higher energy density phosphate systems. This study provides a clear direction of travel for LMFP development and clarifies the challenge to LMFP manufacturing to design morphologies suitable for high compaction (such as bi‐modal particle size distributions), and to the calendaring process to enable sufficiently high levels of pressure.

## Author Contributions

**Rebecca Sellers**: investigation, writing – original draft, data curation, conceptualization. **Daniela Proprentner**: investigation, conceptualization. **Amir Dahari**: writing – review and editing, conceptualization. **Steve Kench**: conceptualization, writing – review and editing, funding acquisition. **Galo Paez‐fajardo**: data curation, formal analysis, writing – review and editing. **Louis Piper**: conceptualization, funding acquisition, writing – review and editing, supervision, resources, project administration. **Gerard Bree**: conceptualization, investigation, funding acquisition, writing – original draft, writing – review and editing, supervision, data curation, project administration.

## Conflicts of Interest

The authors declare no conflicts of interest.

## Supporting information




**Supporting File**: advs78510‐sup‐0001‐SuppMat.docx.

## Data Availability

The data that support the findings of this study are available from the corresponding author upon reasonable request.
