# Supplementary material for: Battery Electrode Engineering Enables Fast Charging LMFP Cells Without Carbon‐Coated Foil
Source: Adv Sci (Weinh). 2026 Oct 26:e78510. Online ahead of print. doi: 10.1002/advs.78510 (PMC13710414; doi:10.1002/advs.78510)
Supplement: Supplementary file 1 [file ADVS-9999-e78510-s001.docx]

Battery Electrode Engineering Enables Fast Charging LMFP Cells Without Carbon-Coated Foil

Rebecca Sellers^1, 2^, Daniela Proprentner^1, 2^, Amir Dahari^3,4^, Steve Kench^3^, Galo Paez-Fajardo^1, 2^, Louis Piper^1, 2,*^, Gerard Bree^1, 2,*^

^1^WMG, University of Warwick, Coventry CV4 7AL, UK

^2^The Faraday Institution, Harwell Science Campus, Didcot OX11 0RA, UK

^3^Polaron, Senna Building, Gorsuch Place, E2 8JF, London, UK

^4^Dyson School of Design Engineering, Imperial College London, London SW7 2DB, UK

*Corresponding authors

# Supplementary Information

|  |  | Pristine | C-coated |  |
| --- | --- | --- | --- | --- |
| Aluminium | Coating Length (m) | 350 | 80 | m |
|  | Carbon thickness (µm) | 0 | 2 | um |
|  | Foil thickness | 15 | 16 | um |
|  | Width | 180 | 180 | mm |
|  |  |  |  |  |
|  | Cost | $ 339.25 | $ 457.70 |  |
|  | Cost / m | $ 0.97 | $ 5.72 |  |
|  | Premium |  |  | 490% |
|  |  |  |  |  |
| Copper | Coating Length (m) | 310 | 40 | m |
|  | Carbon thickness (µm) | 0 | 2 | um |
|  | Foil thickness | 9 | 9 | um |
|  | Width | 180 | 180 | mm |
|  |  |  |  |  |
|  | Cost | $ 735.77 | $ 570.40 |  |
|  | Cost / m | $ 2.37 | $ 14.26 |  |
|  | Premium |  |  | 501% |

Table S1: Cost of carbon coated compared with pristine aluminium and copper foils. Data was taken from the MTI online shop.[1] Note that these prices represent small-scale lab purchases (e.g. 350 m rolls) and do not reflect scaled manufacturing costs..

**Quantifying electrode density and porosity**

Electrode density was calculated utilising measured coat weight and electrode thickness, via:

$$Electrode density (g cm^{-3})= \frac{Coat Weight (g m^{-2})}{Electrode thickness (\mu m)}$$

Porosity was then calculated by comparison of the observed density, with the true density of the electrode formulation:

$$Porosity \left( \% \right)=1- \frac{Electrode density (g {cm}^{-3})}{Formulation true density (g {cm}^{-3})}$$

Where:

$$Formulation true density=\left( Component A true density x mass fraction \right)+\left( Component B true density x mass fraction \right)+\ldots+(Component N true density x mass fraction)$$

For example:

Electrode formulation = 93 : 3.5 : 3.5, LMFP : PVDF : CB (mass ratio)

True densities: LMFP = 3.5 g cm^-3^, PVDF = 1.78 cm^-3^, CB = 2.0 cm^-3^

$$Formulation true density=\left( 93\% x 3.5 \right)+\left( 3.5\% x 1.78 \right)+\left( 3.5\% x 2.0 \right)= 3.38 g cm^{-3}$$

Observed thickness = 60 μm, observed coat weight = 135 g m^-2^

$$Electrode Density= \frac{135 gm^{-2}}{60 \mu m}= 2.25 g cm^{-3}$$

$$Electrode Porosity=1- \frac{2.25 g {cm}^{-3}}{3.38 g {cm}^{-3}}=33 \%$$

Figure S1: Ex-situ HAXPES and XAS of LMFP electrodes to verify material evolution during delithiation. (a) Fe 2p, (b) O 1s, (c) C 1s and (d) Fe L-edge, obtained in the pristine (lithiated) and 100 % SOC (delithiated) states. The delithiated electrode sample was harvested from a half coin cell charged to 4.5 V vs Li^+^/Li. Measurements were carried out Diamond Light Source beamline I09. The results demonstrate changes in line with expected behaviour for LMFP i.e. Fe oxidation from Fe2^+^ to Fe3^+^, along with the presence of carbon in multiple forms (particle coating, carbon black, PVDF binder). Additionally, subsurface oxygen in the LMFP structure exhibits little evolution indicating negligible surface deterioration.

Figure S2: Long-term cycling of an LMFP \\ Graphite pouch cell at C/3, in the range 2.5–4.3 V. This was performed to validate the quality of the LMFP material utilized in this study. The pouch cell format was used for extended cycling to address coin-cell limitations (water ingress) and provide a more industrially relevant environment.

Figure S3: Comparison of XRD spectra of LMFP electrodes in the uncalendared (1.75 g cm^-3^) and calendered state (2.50 g cm^-3^). No evolution peak position was apparent, nor were any new peaks observed, indicating the maintenance of the pure LMFP phase.

Figure S4: Rate capability test of LMFP \\ Graphite full coin cells, comparing (a) specific capacity of pristine and C-coated Al at two LMFP densities, (b) volumetric capacity of pristine Al over 4 densities, (c) specific and (d) volumetric capacity of C-coated Al over the full density range. Shown here are the full 5 cycles at each rate.

Figure S5: Analysis of cell-to-cell variation. Data shown is mean and standard deviation of 3 samples per condition. P-values > 0.05 indicate no statistically significant difference between pristine and C-coated samples, except at final C/3 cycles.

Figure S6. Voltage profiles of LMFP (2.5 g cm^-3^), at C/3 (a) before and (b) after rate capability testing. The C-coated cell exhibited a greater level of Li inventory loss after the test.

Figure S7: Comparison of LMFP \\ Gr cells coated on pristine and C-coated Al foils to two densities, measured during a rate capability test with performance expressed volumetrically.

Figure S8: Cross-sectional SEM images, collected with the backscattered detector, of LMFP electrodes coated on Al foil, and calendered to four densities.

Figure S9: A comparison of voltage profiles of LMFP \\ Gr cells of density 1.95 g cm^-3^, taken from the rate test (2.5 – 4.3 V, black) and the first cycle of the lifetime test (3.0 – 4.3 V, red).

Figure S10: Capacity and coulombic efficiency as a function of cycle number during extended cycling of LMFP\graphite cells of varying LMFP electrode density. LMFP electrodes on (a) pristine and (b) C-coated foils are shown.

Figure S11: Specific energy density of LMFP \\ Gr full coin cells as a function of cycle number over 300 cycles at C/3. Consideration of discharge energy (as opposed to just specific capacity) accounts for the voltage decay mechanism often exhibited by LMFP cells.

Figure S12: Voltage profiles of LMFP \\ Gr full coin cells during cycling at C/3 for 300 cycles.

Figure S13: Long-term cycling at C/3 of LMFP\\Gr full coin cells of varying LMFP electrode density, showing only the C/20 diagnostic cycles.

Figure S14: Cross-sectional SEM image of LMFP electrode of density 2.25 g cm^-3^, harvested from a coin cell which had been aged for 300 cycles.

Figure S15: A comparison of the solid tortuosity factor of a pristine LMFP electrode of density 2.25 g cm^-3^, and a similar electrode harvested from a coin cell aged for 300 cycles. This quantity was determined based on segmentation of cross-sectional SEM images, with analysis software (Polaron AI).

Figure S16: CV step length for various densities as a function of C-rate.

1.	*MTI Corporation Online Shop*. [cited 2026 26/08/2026]; Available from: <https://mtixtl.com/en-euea>.
